# The Value of HALP Score in Predicting Adverse In-Hospital Clinical Outcomes in Patients Undergoing Transcatheter Aortic Valve Replacement

**DOI:** 10.3390/diagnostics16020276

**Published:** 2026-01-15

**Authors:** Ömer Faruk Çiçek, Mustafa Çetin, Ali Palice

**Affiliations:** 1 Department of Cardiology, Mehmet Akif Inan Education and Research Hospital, Sanliurfa 63300, Turkey; 2 Akçakale State Hospital, Sanliurfa 63500, Turkey

**Keywords:** transcatheter aortic valve replacement, HALP score, aortic stenosis

## Abstract

**Background**: Transcatheter aortic valve replacement (TAVR) is widely used in patients with severe aortic stenosis. The HALP (hemoglobin, albumin, lymphocyte, and platelet) score is an easily obtainable composite index that reflects nutritional status and systemic inflammation. **Methods**: In this single-center retrospective study, 140 patients who underwent TAVR between 1 April 2021, and 31 October 2024, were included. Patients were stratified according to the median HALP score (32.65) into low (<32.65)- and high (≥32.65)-HALP groups. In-hospital outcomes were mortality, bleeding requiring transfusion of >5 units of red blood cells, acute kidney injury (AKI), need for mechanical ventilation >24 h, and length of hospital stay. Associations between the HALP score and clinical outcomes were evaluated using multivariable regression analyses, and the discriminatory performance of HALP was assessed using receiver operating characteristic (ROC) curves. **Results**: Patients with low HALP scores had higher rates of in-hospital mortality (11.4% vs. 4.2%; *p* = 0.002), bleeding (28.6% vs. 5.7%; *p* < 0.001), AKI (11.4% vs. 2.9%; *p* < 0.001), and need for mechanical ventilation >24 h (25.7% vs. 14.4%; *p* = 0.002), as well as longer hospital stay (4.82 ± 1.50 vs. 3.62 ± 1.94 days; *p* = 0.001) compared with the high-HALP group. In multivariable models, a lower HALP score remained independently associated with all adverse in-hospital outcomes. ROC analysis showed good discriminatory ability of the HALP score for mortality (area under the curve [AUC] = 0.816; cut-off = 20.16), bleeding (AUC = 0.798; cut-off = 24.94), AKI (AUC = 0.737; cut-off = 26.21), and need for mechanical ventilation (AUC = 0.735; cut-off = 27.36). **Conclusions**: The HALP score is independently associated with adverse in-hospital clinical outcomes in patients undergoing TAVR and may serve as a simple and practical tool for early risk stratification in this population.

## 1. Introduction

Transcatheter aortic valve replacement (TAVR) has emerged as the standard of care for patients with severe aortic stenosis and has effectively replaced surgical aortic valve replacement (SAVR) in eligible patients [1]. Advancements in device technology, interventional techniques, and peri-procedural care have significantly improved clinical outcomes following TAVR [2]. Moreover, contemporary registries and randomized studies report in-hospital or 30-day mortality rates generally below 3–5%, along with reductions in major procedural complications and a trend toward shorter length of hospital stay compared with earlier TAVR eras [3,4,5,6]. However, clinically relevant in-hospital adverse events remain frequent in high-risk patients, underscoring the need for improved early risk stratification [3,7]. Although TAVR is less invasive than surgical aortic valve replacement, close hemodynamic and intensive care monitoring remain essential [3,4]. Furthermore, shorter hospital stays have been associated with improved outcomes and lower health-care costs, highlighting the importance of early discharge when clinically appropriate [5].

Current guidelines recommend assessing frailty before TAVR, as frailty is a strong determinant of post-procedural recovery [8,9]. Serum albumin is a well-known marker of nutritional status and has been associated with frailty and adverse outcomes in patients undergoing TAVR [10]. Risk stratification commonly incorporates scores such as the logistic European System for Cardiac Operative Risk Evaluation (EuroSCORE) and the Society of Thoracic Surgeons (STS) score; however, these systems require numerous clinical inputs and may not fully reflect inflammatory or nutritional burden. Given the high inflammatory and nutritional burden among TAVR candidates, composite indices integrating hematologic, nutritional, and inflammatory parameters may provide additional prognostic insight beyond conventional risk scores [11,12].

The hemoglobin-albumin-lymphocyte-platelet (HALP) score—comprising hemoglobin, albumin, lymphocyte count, and platelet count—is a simple, readily measurable marker of nutritional status and systemic inflammation. It has demonstrated prognostic value in several clinical conditions, including cancer, acute heart failure, stroke, and acute ST-segment elevation myocardial infarction [12,13,14,15]. Lymphocytes modulate inflammatory signaling pathways, while increased platelet activity contributes to thromboinflammation and endothelial dysfunction.

In addition to anemia and hypoalbuminemia, lymphopenia is frequently observed in patients undergoing aortic valve replacement and may reflect chronic systemic inflammation, immunosenescence, and stress-related immune dysregulation [16]. Such immune alterations have been associated with adverse outcomes after aortic valve replacement, supporting the biological rationale for incorporating lymphocyte count into the HALP score [14,16,17]. Additionally, hypoalbuminemia and anemia are well-established indicators of malnutrition and heightened inflammatory stress [14,15].

Despite increasing interest in composite inflammatory–nutritional indices for risk stratification in patients undergoing transcatheter aortic valve replacement [2,10], the prognostic value of the hemoglobin-albumin-lymphocyte-platelet (HALP) score in this population has not yet been investigated. Therefore, a clear gap exists regarding whether this easily obtainable index can predict early adverse clinical outcomes after TAVR. We hypothesized that lower HALP scores, reflecting poorer nutritional status and heightened systemic inflammation, would be associated with an increased risk of in-hospital adverse outcomes following TAVR. Accordingly, the objective of this study was to evaluate the association between the HALP score and in-hospital mortality, bleeding, acute kidney injury, need for prolonged mechanical ventilation, and length of hospital stay in patients undergoing TAVR.

## 2. Materials and Methods

### 2.1. Study Population

Between 1 April 2021, and 31 October 2024, patients who underwent transcatheter aortic valve replacement (TAVR) at the Department of Cardiology, Mehmet Akif İnan Training and Research Hospital (Sanliurfa, Turkey), were retrospectively screened. After application of the eligibility criteria, a total of 140 patients (*n* = 140) were included in the final analysis. Inclusion criteria were patients aged 18 years or older who underwent TAVR. Patients with acute infections (*n* = 4), hematologic diseases (*n* = 5), end-stage hepatic failure (*n* = 4), or active malignancy (*n* = 2) were excluded from the study. Approval of the Local Ethics Board was obtained for the study protocol (Approval No: HRÜ/24.18.41). Artificial intelligence-enabled technologies were not used during data collection, statistical analysis, or interpretation of the results.

### 2.2. Clinical and Laboratory Evaluation

The patients’ demographic data (sex, age, body mass index [BMI]), accompanying diseases (hypertension, diabetes mellitus, coronary artery disease, atrial fibrillation), previous cardiac surgery, New York Heart Association (NYHA) functional class, left ventricular ejection fraction, valve type, and risk scores (logistic EuroSCORE I and Society of Thoracic Surgeons [STS] score) were obtained from the hospital’s electronic medical records. Complete blood counts were analyzed using the Beckman Coulter Hematology Analyzer LH780 (Beckman Coulter, Miami, FL, USA), and serum biochemical parameters were analyzed using the Beckman Coulter Chemistry Analyzer AU680 (Beckman Coulter, Brea, CA, USA). Hematologic and biochemical parameters from venous blood samples collected at the time of the patients’ first hospital admission were recorded. The HALP score was calculated from admission laboratory values using the following formula—hemoglobin (g/L) × albumin (g/L) × lymphocyte count (×10^9^/L) ÷ platelet count (×10^9^/L)—after unit harmonization. Hemoglobin values were converted from g/dL to g/L before HALP calculation. Lymphocyte and platelet counts reported as 10^3^/µL are equivalent to 10^9^/L and were used accordingly in the HALP formula. Patients were then stratified into two groups based on the median HALP value (32.65): low HALP (<32.65) and high HALP (≥32.65).

### 2.3. Endpoints

The analysis focused on five different in-hospital endpoints: mortality, bleeding events, acute kidney injury (AKI), mechanical ventilation exceeding 24 h, and length of hospital stay. In-hospital mortality was defined as death occurring during the procedure or within 30 days of discharge. Bleeding events were defined as the requirement for transfusion of more than 5 units of red blood cells. Acute kidney injury was defined as a ≥50% increase in serum creatinine levels compared to baseline values after the procedure. Mechanical ventilation exceeding 24 h was defined as the need for invasive mechanical ventilation due to pulmonary edema or hypoxia lasting longer than 24 h. Length of hospital stay was defined as the number of days from the TAVR procedure to discharge. For the estimated logistic EuroSCORE I (European System for Cardiac Operative Risk Evaluation), all fields were populated whenever possible; when the item “critical preoperative state” could not be determined, a non-critical preoperative state was assumed, resulting in a best-case scenario.

### 2.4. Statistical Analysis

Data analysis was performed using the Statistical Package for the Social Sciences (SPSS) software, version 24 (IBM Corp., Armonk, NY, USA). Continuous variables are expressed as mean ± standard deviation or median (interquartile range), depending on distribution, and categorical variables as frequency and percentage. Normality of distribution was assessed using the Shapiro–Wilk test, and homogeneity of variances was assessed using the Levene test. For comparisons between the two HALP groups, the independent-samples *t*-test or Mann–Whitney U test was used, as appropriate. At the same time, categorical variables were compared with the chi-square test.

Missing data accounted for fewer than 5% of all variables and were handled using complete-case analysis without imputation. A post hoc power analysis based on the observed difference in composite adverse outcomes between the low- and high-HALP groups demonstrated approximately 80% power at a two-sided alpha level of 0.05.

Associations between the HALP score and clinical outcomes were evaluated using multivariable regression analyses. Covariates were selected based on clinical relevance, and all covariates were entered simultaneously (enter method) into the multivariable models. Binary outcomes (mortality, bleeding, AKI, and mechanical ventilation > 24 h) were analyzed using logistic regression models, and results are presented as regression coefficients (β) with corresponding *p* values (for logistic regression models, β coefficients correspond to log-odds). Length of hospital stay was analyzed using linear regression.

Regression model performance was assessed using R^2^ statistics, and all results were reported with corresponding β coefficients and *p* values. The predictive performance of the HALP score for adverse in-hospital outcomes was evaluated using receiver operating characteristic (ROC) curve analysis, and the area under the curve (AUC) with its 95% CI was calculated. The optimal cut-off values were determined using the Youden index. A two-sided *p* < 0.05 was considered statistically significant.

## 3. Results

**Figure 1:**
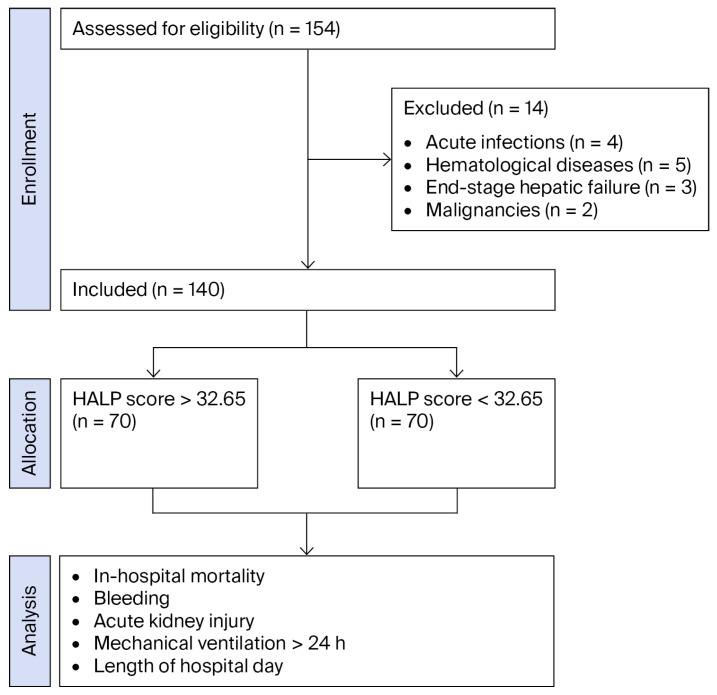
Flow diagram of patient recruitment, exclusions, and final study population.

A total of 154 patients were assessed for eligibility. A total of 14 patients were excluded according to predefined exclusion criteria, and 140 patients were included in the final analysis (Figure 1). The baseline demographic and laboratory characteristics of the 140 patients included in the study are presented in Table 1. Patients were stratified into two groups based on the median HALP value of 32.65, yielding 70 patients in the low-HALP (<32.65) group and 70 in the high-HALP (≥32.65) group. There were no significant differences between the groups regarding sex, body mass index (BMI), hypertension, diabetes mellitus, coronary artery disease, previous cardiac surgery, atrial fibrillation, left ventricular ejection fraction, or the use of self-expandable valves.

In contrast, patients in the low-HALP group had significantly higher logistic EuroSCORE I values and a higher proportion of NYHA class III–IV. Hemoglobin, albumin, and lymphocyte levels were significantly higher in the high-HALP group (all *p* < 0.001), whereas platelet counts were significantly higher in the low-HALP group (*p* < 0.001).

In-hospital adverse outcomes for both groups are shown in Table 2. Patients in the low-HALP group experienced significantly higher rates of in-hospital mortality (11.4% vs. 4.2%; *p* = 0.002), bleeding (28.6% vs. 5.7%; *p* < 0.001), acute kidney injury (11.4% vs. 2.9%; *p* < 0.001), and mechanical ventilation > 24 h (25.7% vs. 14.4%; *p* = 0.002). The length of hospital stay was also significantly longer in the low-HALP group (4.82 ± 1.50 vs. 3.62 ± 1.94 days; *p* = 0.001).

Multivariable regression analyses assessing the association between the HALP score and clinical outcomes are summarized in Table 3. After adjustment for relevant covariates, lower HALP scores remained independently associated with increased mortality, bleeding, acute kidney injury, the need for mechanical ventilation for >24 h, and longer hospitalization. Specifically, the HALP score independently predicted mortality (β = −0.46; *p* < 0.001), bleeding (β = −0.39; *p* = 0.002), acute kidney injury (β = −0.35; *p* = 0.01), mechanical ventilation >24 h (β = −0.33; *p* = 0.02), and length of hospital stay (β = −0.29; *p* = 0.03).

**Table 3:** Multivariable regression models evaluating predictors of adverse in-hospital outcomes.

Variables	In-Hospital Mortality		Bleeding		Acute Kidney Injury		Mechanical Ventilation		Length of Hospital Stay (Days)	
	β	*p*	β	*p*	β	*p*	β	*p*	β	*p*
HALP score	−0.46	<0.001	−0.39	0.002	−0.35	0.01	−0.33	0.02	−0.29	0.03
Age	0.10	0.91	0.05	0.64	0.03	0.71	0.04	0.59	0.03	0.97
Sex (female)	0.13	0.13	0.19	0.06	0.27	0.04	0.04	0.61	0.18	0.06
Diabetes mellitus	0.10	0.21	0.10	0.20	0.03	0.72	0.13	0.11	0.09	0.24
Hypertension	0.12	0.14	0.04	0.58	0.11	0.16	0.09	0.22	0.21	0.01
Coronary artery disease	0.08	0.34	0.11	0.15	0.08	0.31	0.11	0.16	0.06	0.94
Atrial fibrillation	0.06	0.39	0.14	0.08	0.07	0.37	0.26	0.04	0.12	0.16

HALP, hemoglobin, albumin, lymphocyte, and platelet. Values are presented as regression coefficients (β) with corresponding *p*-values. All covariates listed were entered simultaneously into the multivariable models.

**Figure 2:**
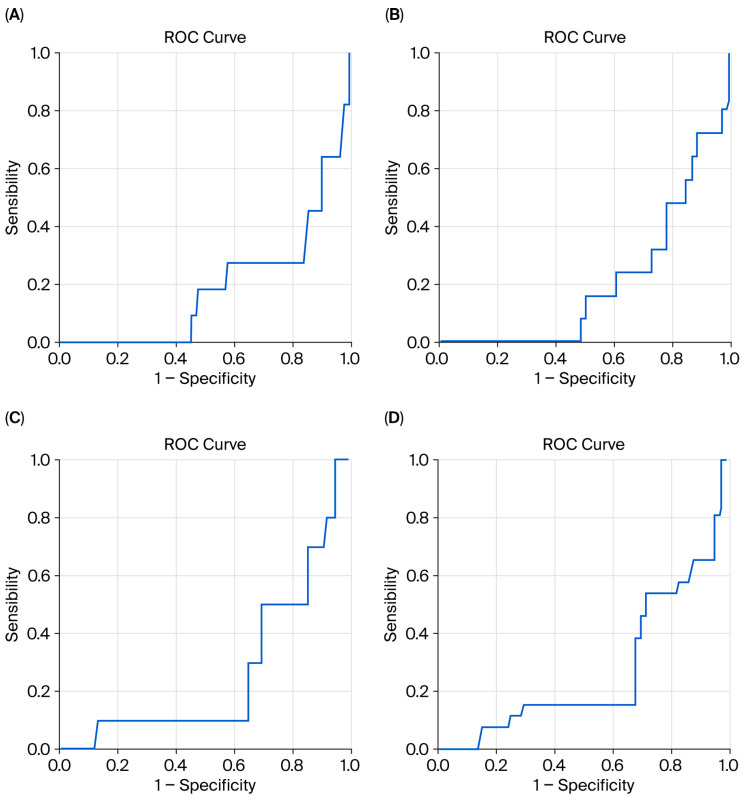
Receiver operating characteristic (ROC) curves demonstrating the discriminatory performance of the HALP score for predicting in-hospital adverse outcomes after transcatheter aortic valve replacement (TAVR): (**A**) in-hospital mortality, (**B**) bleeding, (**C**) acute kidney injury, and (**D**) mechanical ventilation >24 h. (Diagonal segments are produced by ties).

Receiver operating characteristic (ROC) curve analyses demonstrated that the HALP score had good discriminatory ability for predicting adverse outcomes (Figure 2 and Table 4). The AUC for predicting mortality was 0.816 (95% CI: 0.692–0.941; *p* = 0.001), with an optimal cut-off value of 20.16. The HALP score also demonstrated strong discrimination for bleeding (AUC = 0.798; 95% CI: 0.715–0.881; *p* < 0.001; cut-off 24.94), acute kidney injury (AUC = 0.737; 95% CI: 0.584–0.890; *p* = 0.013; cut-off 26.21), and the need for mechanical ventilation > 24 h (AUC = 0.735; 95% CI: 0.626–0.843; *p* < 0.001; cut-off 27.36). These findings indicate that the HALP score demonstrates a significant ability to discriminate and predict in-hospital adverse clinical outcomes following TAVR.

## 4. Discussion

The principal finding of this study is that a lower HALP score was independently associated with a higher risk of multiple adverse in-hospital outcomes, including mortality, bleeding, acute kidney injury, prolonged mechanical ventilation, and longer hospital stay in patients undergoing transcatheter aortic valve replacement. These findings suggest that an integrated inflammatory–nutritional burden, as reflected by the HALP score, plays a critical role in early vulnerability following TAVR and may complement conventional risk stratification tools.

The HALP score was originally introduced to assess survival in patients with gastric cancer [18] and has since been used as a prognostic indicator in several malignancies and in ischemic stroke [14]. The HALP score reflects both nutritional and inflammatory status by integrating hemoglobin, albumin, lymphocyte, and platelet levels. Previous studies have consistently shown that lower HALP levels are associated with poorer survival across different clinical conditions [11,14]. To our knowledge, our study is the first to evaluate the predictive value of the HALP score for in-hospital adverse clinical outcomes in patients undergoing TAVR.

Hemoglobin is one of the key components of the HALP score. Anemia is associated with adverse outcomes not only after TAVR but also in a broad range of cardiovascular diseases and interventional cardiac procedures [19,20,21]. Possible mechanisms include compensatory increases in cardiac output, impaired systemic oxygen delivery, tachycardia, and myocardial ischemia [22,23]. A recent analysis from the Optimized Transcatheter Valvular Intervention Registry demonstrated that severe anemia (hemoglobin <10.5 g/dL) was independently associated with significantly higher all-cause and cardiovascular mortality [24]. Consistent with these data, our study showed that lower hemoglobin levels—reflected by a lower HALP score—were associated with worse in-hospital outcomes after TAVR.

Albumin, another major component of the HALP score, plays essential physiological roles and is the predominant protein within the intravascular space. Hypoalbuminemia is a well-recognized predictor of poor outcomes in patients undergoing TAVR. In the study by Yamamoto et al., low baseline albumin levels were associated with increased mortality after TAVR, suggesting that albumin may serve as a useful marker for pre-procedural risk evaluation [10]. In our study, albumin levels were significantly lower in patients with low HALP scores, underscoring the relationship between malnutrition, systemic inflammation, and early complications. Albumin functions as a negative acute-phase reactant, and its synthesis may be inhibited by inflammatory cytokines such as interleukin-6, leading to reduced serum concentrations during systemic inflammation [25].

Lymphocyte count is another component of the HALP score. Analyses from the PARTNER registry demonstrated that an elevated neutrophil-to-lymphocyte ratio (NLR) was independently associated with increased mortality and rehospitalization after TAVR or surgery [16]. Lymphopenia may therefore be indicative of impaired immune response and increased inflammatory burden. Platelets also contribute to the prognostic value of the HALP score by playing critical roles in coagulation, inflammation, endothelial dysfunction, tissue repair, and wound healing [26]. Conversely, high platelet counts have been linked to vascular complications, major bleeding, and in-hospital mortality following TAVR [27], supporting the biological plausibility of the HALP score. Although hemoglobin and albumin are major components of the HALP score, the more pronounced relative differences observed in lymphocyte and platelet counts between the groups may suggest that inflammatory and thromboinflammatory pathways contribute substantially to the separation of HALP scores.

Our findings demonstrate that patients with low HALP scores experienced significantly higher rates of in-hospital mortality, bleeding, acute kidney injury, prolonged mechanical ventilation, and longer hospital stays. The HALP score thus represents an integrated biomarker that reflects hematologic, nutritional, and inflammatory vulnerability. These findings suggest that patients with low HALP scores may have reduced physiological reserve, rendering them more susceptible to the procedural and perioperative stress associated with TAVR. Previous studies have likewise shown that low lymphocyte and albumin levels, along with high platelet counts, are associated with adverse outcomes such as infection, bleeding, vascular complications, and prolonged hospitalization [17]. However, our study did not include subgroup analyses (e.g., by age, sex, or comorbidity burden); therefore, whether the predictive value of the HALP score varies across different patient subgroups remains unknown.

Recent studies have increasingly emphasized the importance of post-procedural medical optimization in improving outcomes after aortic valve replacement. In particular, guideline-directed medical therapy for heart failure, including sodium–glucose cotransporter 2 (SGLT2) inhibitors, has emerged as a key component of contemporary management. SGLT2 inhibitors such as dapagliflozin have been shown to reduce all-cause mortality and heart failure hospitalizations after transcatheter aortic valve replacement, while also being associated with improved cardiac remodeling, a lower risk of bioprosthetic valve failure, and a potential mitigation of kidney injury, especially in high-risk and diabetic patient populations [28]. Although the present study did not evaluate post-TAVR pharmacological therapies, these emerging data highlight that inflammatory–nutritional status, as reflected by the HALP score, and optimized medical therapy may act synergistically to influence early and late outcomes after TAVR.

The HALP score may serve as a simple and practical prognostic tool to support pre-procedural risk stratification in TAVR candidates. Although established risk models such as the logistic EuroSCORE-1, EuroSCORE-2, and the Society of Thoracic Surgeons (STSs) score remain essential for evaluating procedural risk, they require multiple clinical parameters and do not fully capture the nutritional or inflammatory burden. In contrast, the HALP score is inexpensive, is easily calculated from routine admission laboratory tests, and may offer additional prognostic insight. Nonetheless, larger, prospective, multicenter studies are needed to validate these findings and further elucidate the role of the HALP score in predicting adverse in-hospital outcomes following TAVR.

## 5. Limitations

This study has several limitations. First, it was conducted at a single center, which may limit the generalizability of the findings and introduce selection bias. Second, the sample size was relatively small, and although a post hoc power analysis suggested adequate statistical power, the results should still be interpreted with caution. Third, our study did not include subgroup analyses (e.g., according to age, sex, or comorbidity burden); therefore, potential variations in the predictive value of the HALP score across different patient populations could not be assessed. In addition, the listed comorbidities represent the most prevalent cardiovascular conditions routinely recorded in the institutional database. Other potentially relevant cardiovascular and non-cardiovascular comorbidities were not systematically available and therefore were not included in the analysis, which may have introduced residual confounding. Finally, the retrospective design may limit the ability to establish causal relationships. Prospective multicenter studies are needed to validate and expand upon these results.

## 6. Conclusions

The HALP score is a simple, practical index that can be easily calculated from routinely obtained laboratory parameters. This study demonstrates that the HALP score may serve as a useful prognostic marker for predicting adverse in-hospital clinical outcomes in patients undergoing transcatheter aortic valve replacement. By providing additional insight into hematologic, nutritional, and inflammatory status, the HALP score may help clinicians identify higher-risk patients before the procedure. Incorporation of the HALP score into routine pre-procedural assessment may therefore support early risk stratification in this population.

## Figures and Tables

**Table 1:** Baseline demographic, clinical, and laboratory characteristics of patients stratified by HALP Score.

	HALP > 32.65 (*n* = 70)	HALP < 32.65 (*n* = 70)	*p*
Sex (female), *n* (%)	34 (48.6%)	38 (54.3%)	0.49
Age (years)	74.05 ± 5.42	78.25 ± 6.83	**<0.001**
BMI (kg/m^2^)	26.89 ± 2.95	26.33 ± 4.32	0.37
Diabetes mellitus *n* (%)	18 (25.7%)	22 (31.4%)	0.45
Hypertension, *n* (%)	24 (34.3%)	32 (45.7%)	0.16
Coronary artery disease, *n* (%)	32 (45.7%)	38 (54.3%)	0.31
Cardiac surgery, *n* (%)	10 (14.3%)	10 (14.3%)	1.00
Atrial fibrillation, *n* (%)	8 (11.4%)	16 (22.9%)	**0.07**
Left ventricular ejection fraction (%)	51.22 ± 8.80	50.14 ± 10.10	**0.49**
Logistic EuroSCORE I	19.59 ± 9.6	23.01 ± 8.19	**0.02**
NYHA functional class III or IV, *n* (%)	28 (40%)	46 (65.7%)	**0.004**
Self-expandable valve, *n* (%)	42 (60%)	30 (42.9%)	**0.062**
Hemoglobin (g/dL)	11.73 ± 1.33	10.77 ± 1.42	**<0.001**
Albumin (g/L)	39.21 ± 2.95	35.30 ± 4.92	**<0.001**
Lymphocyte (10^3^/µL)	2.38 ± 0.79	1.60 ± 0.53	**<0.001**
Platelet (10^3^/µL)	224.94 ± 61.15	293.77 ± 80.91	**<0.001**
HALP score	49.79 ± 17.56	20.97 ± 6.24	**<0.001**

The values indicated in bold are statistically significant (*p* < 0.05). NYHA, New York Heart Association functional classification; HALP, hemoglobin, albumin, lymphocyte, and platelet.

**Table 2:** In-Hospital adverse clinical outcomes according to HALP score groups.

Adverse Clinical Outcomes	HALP > 32.65 (*n* = 70)	HALP < 32.65 (*n* = 70)	*p*
In-hospital mortality, *n* (%)	3 (4.2%)	8 (11.4%)	**0.002**
Bleeding, *n* (%)	4 (5.7%)	20 (28.6%)	**<0.001**
Acute kidney injury, *n* (%)	2 (2.9%)	8 (11.4%)	**<0.001**
Mechanical ventilation > 24 h, *n* (%)	8 (14.4%)	18 (25.7%)	**0.002**
Length of hospital stay (days), mean ± SD	3.62 ± 1.94	4.82 ± 1.50	**0.001**

The values indicated in bold are statistically significant (*p* < 0.05). HALP, hemoglobin, albumin, lymphocyte, and platelet.

**Table 4:** Receiver operating characteristic (ROC) analysis of HALP score for predicting in-hospital adverse outcomes.

Adverse Clinical Outcomes	AUC	95% CI	Cut-Off	*p*
In-hospital mortality	0.816	0.692–0.941	20.16	0.001
Bleeding	0.798	0.715–0.881	24.94	<0.001
Acute kidney injury	0.737	0.584–0.890	26.21	0.013
Mechanical ventilation > 24 h	0.735	0.626–0.843	27.36	<0.001

AUC, area under the curve; CI, confidence interval.

## Data Availability

The data presented in this study are available on request from the corresponding author. The data are not publicly available due to privacy and ethical restrictions.
